# Secondary psychotic features in refugees diagnosed with post-traumatic stress disorder: a retrospective cohort study

**DOI:** 10.1186/s12888-016-1166-1

**Published:** 2017-01-05

**Authors:** Mette Nygaard, Charlotte Sonne, Jessica Carlsson

**Affiliations:** Competence Centre for Transcultural Psychiatry, Mental Health Centre Ballerup, Maglevænget 2, 2750 Ballerup, Denmark

**Keywords:** Complex PTSD, Psychotic symptoms, PTSD, PTSD-SP, Refugees

## Abstract

**Background:**

A substantial amount of refugees (10–30%) suffer from Post-Traumatic Stress Disorder (PTSD). In Denmark there are different facilities specialised in psychiatric treatment of trauma-affected refugees. A previously published case report from such a facility in Denmark shows that some patients suffer from secondary psychotic symptoms alongside their PTSD.

The aim of this study was to illustrate the characteristics and estimate the prevalence of psychotic features in a clinical population of trauma-affected refugees with PTSD.

**Methods:**

Psychiatric records from 220 consecutive patients at Competence Centre for Transcultural Psychiatry (CTP) were examined, and all the PTSD patients were divided into two groups; one group with secondary psychotic features (PTSD-SP group) and one without (PTSD group). A categorisation and description of the secondary psychotic features was undertaken.

**Results:**

One hundred eighty-one patients were diagnosed with PTSD among which psychotic symptoms were identified in 74 (40.9, 95% CI 33.7–48.1%). The majority of symptoms identified were auditory hallucinations (66.2%) and persecutory delusions (50.0%). There were significantly more patients diagnosed with enduring personality change after catastrophic experience in the PTSD-SP group than in the PTSD group (*P* = 0.009). Furthermore the PTSD-SP group included significantly more patients exposed to torture (*P* = 0.001) and imprisonment (*P* = 0.005).

**Conclusion:**

This study provides an estimation of PTSD-SP prevalence in a clinical refugee population with PTSD. The study points to the difficulties distinguishing psychotic features from flashbacks and the authors call for attention to psychotic features in PTSD patients in order to improve documentation and understanding of the disorder.

## Background

Disasters and wars take place continuously. An inevitable consequence is that people have to flee their homes and become refugees. The United Nations 1951 Refugee Convention defines a refugee as “*someone who is unable or unwilling to return to their country of origin owing to a well-founded fear of being persecuted for reasons of race, religion, nationality, membership of a particular social group, or political opinion*” [1]. This implies that most refugees have been exposed to traumatic events such as persecution, imprisonment, torture, violence and loss of family members, and additionally traumas due to fleeing from their countries of origin.

Post-Traumatic Stress Disorder (PTSD) is a mental disorder that arises from the experience of a traumatic event of an exceptionally threatening character, which is likely to cause distress in almost anyone as defined in ICD-10 [2]. PTSD is commonly associated with depression, anxiety disorders, alcohol/drug dependency and personality disorders [3–5]. In addition to the PTSD diagnosis, ICD-10 has introduced a separate diagnostic entity called *enduring personality change after catastrophic experience* (DF62.0) that includes a hostile understanding of the surroundings, social withdrawal and a sensation of being under constant threat.

Studies on the prevalence of PTSD in refugees and post-conflict populations worldwide have found a prevalence between 10–30% [6, 7]. Refugees have often been exposed to prolonged and repeated traumas and have been described to present with a more complex symptomatology than is captured by the ICD-10/DSM-V diagnosis of PTSD [6, 8, 9]. In the literature, a diagnostic entity called complex PTSD has long been discussed as survivors of prolonged and repeated trauma often report additional symptoms alongside their PTSD [10]. The symptoms reported in the literature are, for example, emotional regulation difficulties, dissociative and psychotic symptoms and somatic distress. The discussion addresses the current PTSD formulations’ ability to capture these diverse clusters of symptoms. Evidence for the support of the diagnosis complex PTSD is mixed and, although it has not been included in DMS-5 or ICD-10 [11], it will most probably be included in ICD-11 [12–15].

PTSD with Secondary Psychotic features (PTSD-SP) has similarly been discussed as a separate diagnostic entity in which PTSD is followed by the appearance of psychotic features. Studies concerning PTSD-SP show that the psychotic features do not exclusively occur in relation to flashbacks, and that they are chronic. The symptoms described primarily consist of hallucinations and delusions, and are generally related to the patients’ trauma [16, 17]. Braakman el al. proposes that 44 to 84% of patients with PTSD have co-morbid major depressive disorder, and that the comorbidity is probably higher in PTSD-SP patients. They suggest that PTSD, major depressive disorder and psychotic symptoms are somehow interconnected [16].

The literature suggests that PTSD-SP patients suffer from higher burden of disease than other PTSD patients, a burden comparable with schizophrenia [16, 18]. According to Braakman et al., the frequency of PTSD-SP within a PTSD population varies between 15 and 64% in different studies, primarily depending on the sampling strategies.

The vast majority of studies concerning PTSD-SP are conducted on war veterans or victims of sexual assault. Studies of PTSD-SP in refugee populations are however needed, as the population differs from other groups of PTSD patients in several ways. Refugees have usually been exposed to more prolonged trauma than previously studied trauma populations and are thus assumedly more likely to develop severe trauma-related psychopathology [6, 9]. Furthermore, unlike war veterans and victims of sexual assault, refugees have to start up a new life in a different setting after the exposure to trauma, which often involves a new environment with a new culture/society/language and a lack of support from surrounding family and friends [19, 20]. Various studies have pointed to factors operating after the trauma, such as social stressors as risk factors for the development of PTSD [21, 22]. Several of these risk factors, such as lack of social support are frequent among refugees and have been found to be related to levels of PTSD and depression in trauma-affected refugees [23, 24].

A case report from the treatment facility where the present study was carried out described several patients with severe psychopathology and secondary psychotic symptoms alongside their PTSD [17]. We suggest that a more thorough understanding of PTSD-SP patients’ symptoms will facilitate treatment that is more targeted towards the symptoms experienced by the patients and consequently will increase the clinical utility of the diagnosis [25].

The aim of the study was therefore to estimate the prevalence and illustrate characteristics of psychotic features in a clinical population of trauma-affected refugees with PTSD by

1. Estimating the prevalence of PTSD-SP in trauma-affected refugees with PTSD based on psychiatric records at a highly specialised treatment facility.
2. Comparing the PTSD-SP group with the PTSD group with regards to gender, the medication at baseline, the prevalence of depression and enduring personality change after catastrophic experience as well as the type of trauma exposure.
3. Providing a description of psychotic features presented in the PTSD-SP group.

Based on the existing literature and the clinical experience at CTP we hypothesised that a significant number of trauma-affected refugees had a more complex psychopathology than is captured by the ICD-10/DSM-V diagnosis of PTSD, and that some of the experienced features would be identified as psychotic. We also hypothesised that type of trauma exposure, comorbid depression and enduring personality change after catastrophic experience would be related to having psychotic features.

In the following manuscript we will use PTSD-SP to describe patients with psychotic features alongside a PTSD diagnosis, although aware that clear diagnostic criteria’s are not available in the literature. We will more thoroughly describe our definition of PTSD-SP in the method section.

## Methods

### The setting

The study was a retrospective cohort study conducted at Competence Centre for Transcultural Psychiatry (CTP). CTP is a part of the Mental Health Services of the Capital Region of Denmark. The clinic is staffed by psychiatrists, medical doctors, psychologists, social workers and interpreters. Patients are referred to CTP by general practitioners, psychiatrists and medical doctors from psychiatric departments.

At CTP the treatment is based on the existing evidence regarding effective treatment for PTSD, the preliminary results from research carried out at CTP and on clinical experience with the target group [20, 26–30]. The duration of the treatment at CTP is approximately six months; it includes a total of ten consultations with a medical doctor, 16 psychotherapy sessions and one to three sessions with a social worker. The psychiatric consultations include psychoeducation as well as pharmacological treatment.

The target group at the clinic at the time of this study were refugees or individuals who had been family reunified with a refugee. To be admitted to treatment patients should be older than 17 years, have a history of exposure to trauma (such as war, persecution, imprisonment or torture), present with trauma-related mental health problems at the initial psychiatric assessment (often PTSD and/or depression according to the ICD-10 criteria), and should be motivated for treatment. Patients were normally excluded from treatment at CTP if they had a present alcohol or drug abuse and if they met the criteria for a primary psychotic disorder (DF2x) or bipolar disorder (DF31x).

The guidelines for pharmacological treatment at CTP during the study period included first-line treatment with sertraline and add-on treatment with mianserin in case of sleep disturbances. The first choice antipsychotic treatment was perphenazine. Perphenazine was offered if sertraline on the highest tolerable dose (maximum 200 mg) failed to reduce the patient’s psychotic symptoms. Since 29th March 2012 perphenazine has been replaced by quetiapine as perphenazine was taken off the Danish pharmaceutical market during the study period.

### Study sample

Only CTP’s target group is invited to an initial psychiatric assessment (please see above “The setting” for a description of the target group). The inclusion criteria for this study were attending an initial psychiatric assessment at CTP from 14th June 2011 to 29th March 2012 and having a discharge diagnosis of PTSD. The only exclusion criterion was not having a PTSD discharge diagnosis. The psychiatric records of all patients (*n* = 220) who attended an initial psychiatric assessment at CTP from 14th June 2011 to 29th March 2012 were therefore screened and 181 individuals with a PTSD discharge diagnosis were identified. The specific period of time was chosen for practical reasons as it was the inclusion period for a randomised trial and therefore the population was well-defined and data for the patient records had been collected systematically [31]. Baseline characteristics (gender, age, country of origin [32], concurrent medicine at the initial psychiatric assessment, diagnosis in the discharge letter and exposure to trauma) were registered for all patients diagnosed with PTSD at discharge. Subsequently one of the researchers, the first author thoroughly assessed the psychiatric records of all 181 patients for PTSD-SP symptoms according to the study definition (please see below). The psychiatric records were written by seven different medical doctors, who worked at CTP during this time, whereof two of the CTP doctors are the second and third authors of this paper. The first author conferred the content of the psychiatric records with the second and third authors when in doubt.

Based on the findings from the psychiatric records, the study population was divided into two groups; a group of patients suffering from PTSD-SP (called PTSD-SP group) and a group of patients suffering from PTSD without secondary psychotic features (PTSD group).

#### Study definition and selection of PTSD-SP group

In this study, the data on psychopathology relied on data already collected by the clinicians in the patient records. PTSD-SP was defined as patients having the PTSD diagnosis (DF43.1) according to the ICD-10 criteria and showing secondary psychotic features that had been registered during the treatment programme at CTP.

First a preliminary assessment was made by the first author. In this process all sentences from the psychiatric assessments where psychotic symptoms were mentioned were transferred to the datasheet. The following definition of PTSD-SP was agreed on by the authors: 1. secondary psychotic features included hallucinations as well as delusions, experienced while awake, 2. experiences described in relation to sleep were not included (hypnogogic/hypnopompic), 3. patients described with intact reality testing were included and 4. only patients with flashbacks described as being connected to psychotic or psychotic-like symptoms were included in the study. Next a second assessment was made by the authors to make sure the PTSD-SP group were within the described definition. Lastly the secondary psychotic features were categorised in groups for description inspired by *Schedules for Clinical Assessment in Neuropsychiatry* (SCAN), part two, section 16–19 [33]. The categories used for description were: hallucinations (including auditory, visual, olfactory and tactile hallucinations) and delusions (persecutory delusions, delusions of control and bizarre/strange delusions).

### Data management and statistics

The study was accepted by the Danish Data Protection Agency (journal no. RHP-2016-013, I-suite no. 0470), and an ethical approval of the study obtained from the Danish Patient Safety Authority (journal no 3-3013-1722/1/, reference NAAN).

The prevalence of PTSD and PTSD-SP were calculated. The PTSD and PTSD-SP groups were compared with a Pearson Chi-Square test concerning gender, medication at baseline (antidepressant, antipsychotics, benzodiazepines, none), comorbid depression or enduring personality change after catastrophic experience (DF62.0) and type of trauma exposure (torture, imprisonment, lived in war zones, lived in refugee camp, soldier in war). Random lack of data was found concerning type of trauma exposure and only patients where data was available were included in the analysis. SPSS, Version 20 was used for descriptive statistics and Pearson Chi-square test with a significance level of 0.05.

## Results

### Estimating the prevalence of PTSD-SP in trauma-affected refugees with PTSD

**Fig. 1:**
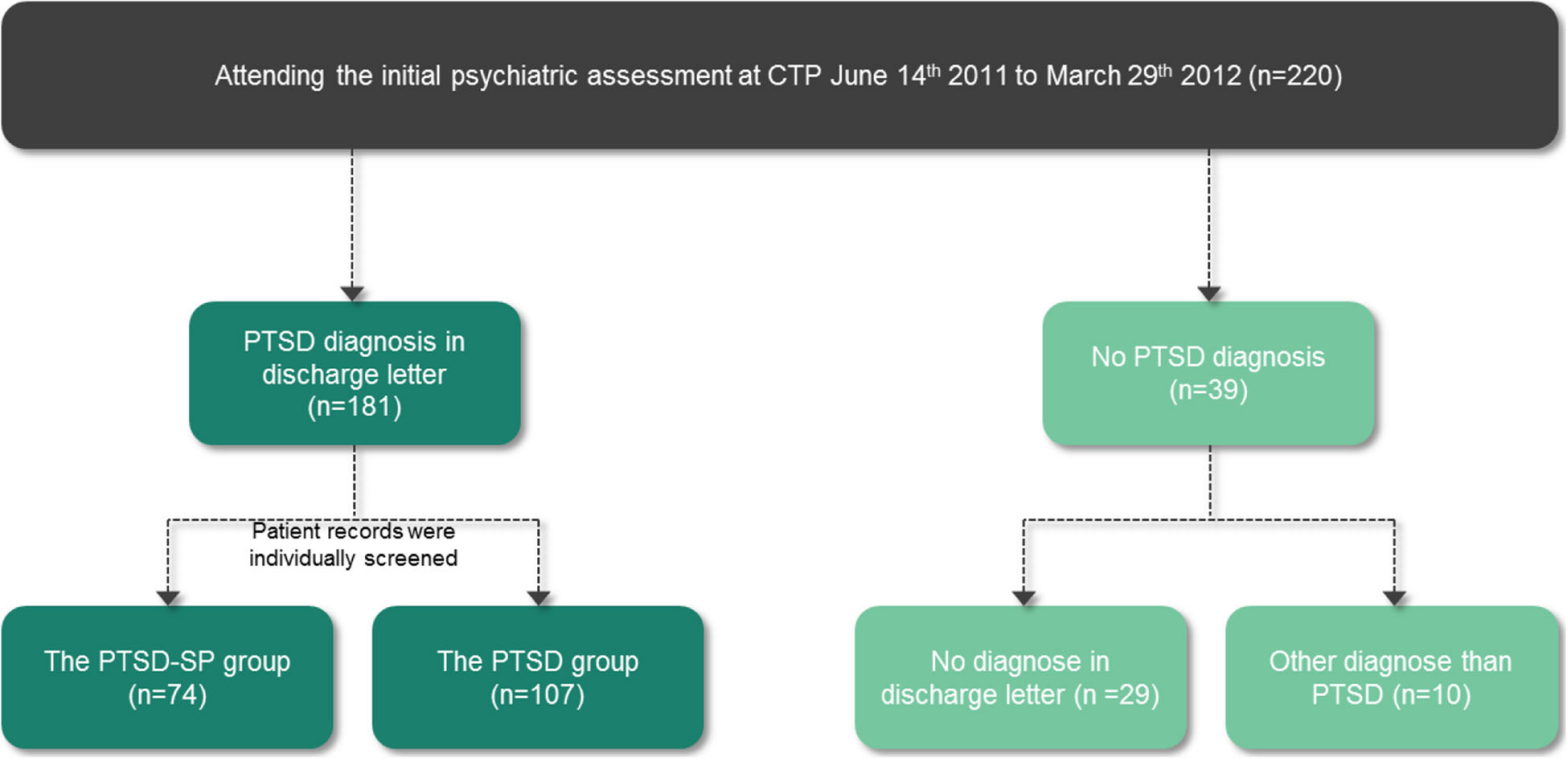
*Selection of the PTSD-SP and the PTSD group*

During the inclusion period 220 patients were referred to CTP. Of those 220, 39 patients did not have a discharge PTSD diagnosis. Among the 39 excluded patients, ten patients presented with another discharge diagnosis than PTSD whereas 29 patients had no discharge diagnosis. For the patients with no discharge diagnosis, seven had not finished their treatment at CTP at the time the study was conducted and for the rest (*n* = 22) the patients were either not the target group of CTP or did not wish treatment and therefore no psychiatric diagnosis had been given after the initial psychiatric assessment. Of the 181 trauma-affected refugees diagnosed with PTSD, 74 (40.9, 95% CI 33.7–48.1%) were classified as having PTSD-SP (see Fig. 1). Two patients were excluded from the PTSD-SP group reasoning lack of description. First patient: *“patients brother tell he is paranoid and suspicious, not further described*”. Second patient: *“feel like two persons, not further described”.*

### Comparing the PTSD-SP group with the PTSD group

The characteristics of the PTSD-SP group and the PTSD group are illustrated in Table 1. The majority of the patients came from South and West Asian countries like Iraq, Lebanon, Iran and Afghanistan.

**Table 1:** Characteristics of the PTSD-SP group (*n* = 74) and the PTSD group (*n* = 107)

		*PTSD-SP*	*PTSD*	
***Characteristics***		***N (%)/mean*** ***(±SD)***	***N (%)/mean*** ***(±SD)***	**Chi-square test**
Male		48 (64.9%)	56 (52.3%)	*P* = 0.094
Female		26 (35.1%)	51 (47.7%)	
Age (3rd May 2013)		44.8 (±8.8)	45.0 (±9.8)	
***Region of origin***	***Countries***	***N (%)***	***N (%)***	
Eastern Asia	China	0	1 (0.94%)	
Southern Asia	Afghanistan, Iran, Sri Lanka	15 (20.3%)	32 (29.9%)	
Western Asia	Iraq, Kuwait, Lebanon, Palestine, Syria, Turkey	45 (60.8%)	55 (51.4%)	
Eastern Europe	Russia	1 (1.35%)	0	
Southern Europe	Albania, Bosnia, Former Yugoslavia	9 (12.2%)	12 (11.2%)	
Eastern Africa	Eritrea, Rwanda, Somalia	2 (2.7%)	4 (3.74%)	
Northern Africa	Algeria, Morocco	1 (1.35%)	1 (0.94%)	
Unknown		1 (1.35%)	2 (1.87%)	
***Comorbidity***	***ICD-10 codes***	***N (%)***	***N (%)***	***Chi-square test***
Depressive disorders	DF32.0, DF32.1, DF32.2, DF32.3, DF32.9, DF33.1, DF33.2, DF33.3, DF33.8, DF33.9	65 (87.8%)	91 (85.1%)	*P* = 0.593
Other affective disorder	DF31.6, DF34.1, DF34.8	0	4 (3.74%)	
Personality change after catastrophic experience	DF62.0	24 (32.4%)	17 (15.9%)	*P* = 0.009*
Mental and behavioural disorders due to psychoactive substance use	DF10.1, DF10.2, DF11.2, DF15.1, DF19.2	2 (2.70%)	3 (2.80%)	
Schizophrenia, Schizotypal and delusional disorders	DF22.9, DF29.9	3 (4.05%)	0	
Other anxiety disorders	DF41.0, DF41.1	3 (4.05%)	5 (4.67%)	
Other personality changes	DF60.0, DF60.1, DF61.0, DF62.1, DF68.0	2 (2.70%)	3 (2.80%)	
No-comorbidity to PTSD		6 (8.11%)	9 (8.41%)	
***Psychiatric medication baseline***	***Generic name***	***N (%)***	***N (%)***	***Chi-square test***
Antipsychotics	flupentixol, chlorprothixen, quetiapin, aripiprazol, olanzapine, risperdon, unspecified	14 (18.9%)	11 (10.3%)	*P* = 0.098
Antidepressants	SSRI, SNRI, TCA, NaSSA, unspecified	35 (47.3%)	46 (43.0%)	*P* = 0.567
Benzodiazepines		10 (13.5%)	24 (22.4%)	*P* = 0.131
No psychopharmacological treatment		30 (40.5%)	50 (46.7%)	*P* = 0.410
***Type of trauma exposure***		***N (%)***	***N (%)***	***Chi-square test***
Exposed to torture *PTSD-SP (n = 69), PTSD (n = 92)*		47 (63.5%)	39 (36.4%)	*P* = 0.001*
Experienced imprisonment *PTSD-SP (n = 69), PTSD (n = 92)*		44 (59.5%)	38 (35.5%)	*P* = 0.005*
Lived in a war zone *PTSD-SP (n = 69), PTSD (n = 91)*		63 (85.1%)	88 (82.2%)	*P* = 0.142
Lived in a refugee camp *PTSD-SP (n = 69), PTSD (n = 85)*		19 (25.6%)	26 (24.3%)	*P* = 0.679
Soldier in war *PTSD-SP (n = 68),PTSD (n = 87)*		23 (31.1%)	23 (21.5%)	*P* = 0.318

* = P < 0.05. ±SD illustrates the standard deviation of the age. Number of patients analysed within each group is displayed as n = xx in “traumaticevents”. The percentages illustrate the amount of patients in the corresponding group

Bold data are the subheadings

There was no significant difference in the gender distribution between the two groups (*P* = 0.094). Patients in both the PTSD group and the PTSD-SP group took a variety of psychopharmacological medication at baseline of the study as illustrated in Table 1. There was no significant difference in medication at baseline between the groups (antipsychotics *P* = 0.098, antidepressant *P* = 0.567, benzodiazepines *P* = 0.131, none *P* = 0.410).

The most frequent comorbid diagnosis in both groups was depression, which was found in 87.8% of patients in the PTSD-SP group and 85.1% in the PTSD group. No significant difference was found between the groups in relation to comorbid depression (*P* = 0.593). The Chi-square test conducted indicated that there were significantly more patients in the PTSD-SP with enduring personality change after catastrophic experience (*P* = 0.009). Three patients from the PTSD-SP group were found to have a DF2x diagnosis in the discharge letter, but PTSD was assessed as the primary diagnosis.

As presented in Table 1; 47 of 74 patients (63.5%) in the PTSD-SP group were exposed to torture as opposed to 39 of 107 patients (36.4%) in the PTSD group. A Chi-square test showed a significant difference between the groups (*P* = 0.001). Similarly, exposure to imprisonment showed a significant difference between the two groups (*P* = 0.005). Chi-square tests were equally conducted concerning whether the patients had been living in war zones (*P* = 0.142), lived in refugee camps (*P* = 0.679) and participated in war as soldiers (*P* = 0.318), but no significant difference between the PTSD-SP group and the PTSD group was found.

### Description of psychotic features presented in the PTSD-SP group

Table 2 shows the distribution of secondary psychotic features in the PTSD-SP group. The most common psychotic symptom observed in the group was auditory hallucinations (66.2%), followed by persecutory delusions (50.0%). Also, visual hallucinations (29.7%) were frequently seen in the PTSD-SP group. More rare symptoms included olfactory (6.8%), tactile hallucinations (8.1%), delusion of control (5.4%) and bizarre delusions (1.4%).

**Table 2:** Psychotic symptoms in PTSD-SP population (*n* = 74)

*Psychotic symptoms*		*N (%)*
Hallucinations	Auditory	49 (66.2%)
	Visual	22 (29.7%)
	Olfactory	5 (6.76%)
	Tactile	6 (8.11%)
Delusions	Persecutory	37 (50.0%)
	Delusion of control	4 (5.41%)
	Bizarre/strange delusion	1 (1.35%)

#### Auditory hallucinations (*n* = 49)

Auditory hallucinations were described in the psychiatric records of 49 (66.2%) of the 74 PTSD-SP patients. Of these 49 patients, 30 had verbal auditory hallucinations, where they heard people speaking or mumbling.

Patients hearing known voices were described in 8 of 30 psychiatric records. The voices were described as coming from e.g. family members; *“can hear ex-husbands voice in the room”* or *“hear and talk to family members”.* Other patients heard voices from their previous perpetrators; *“hearing the voice of the perpetrator in jail”*, the auditory hallucinations were sometimes described as being connected to flashback experiences; *“Hearing flashback saying: “hide”! The patient also hears men from the torture”*.

More unspecific voices or people mumbling were described in 19 psychiatric records. Examples of unspecific voices include; *“hearing people talking together”*, *“hearing voices from angry men”* or *“hearing children calling”*. The voices can also have character of second person auditory hallucinations, e.g.; *“hearing voices calling him”* or *“voice telling him he should shave his hair off, he did that”.* Three patients heard voices with a more mood-congruent content; *“hearing voices saying she is not worth anything (inside her head, know they are not real)”* or *“hearing reproachful voices from lost friends”*.

Six patients heard non-verbal sounds, such as; *“patient can hear a flute playing”* or *“sounds from airplanes (inside the head)”*. Additionally, nine patients in the PTSD-SP group were described as having auditory hallucinations, or possible auditory hallucinations, in their record but these were not further described.

#### Visual hallucinations (*n* = 22)

Visual hallucinations were described in 22 (29.7%) of the 74 patients’ psychiatric records. In 11 records, patients were described as seeing formed hallucinations. These hallucinations were either family members (*n* = 5), for example; *“see father, know it’s not real”, “get visits from brother, mother and neighbour’s dog”*, people from the traumatic events (*n* = 3), for example; *“see men responsible for torture approximately every second day”* or other things such as *“see dangerous animals showing their teeth”*. Other patients (*n* = 4) saw unformed hallucinations such as shadows and ghosts, and yet others (*n* = 7) were described as having visual hallucinations related to the trauma or visual hallucinations mostly in darkness but not further described.

#### Olfactory hallucinations (*n* = 5)

Olfactory hallucinations were described in five (6.8%) of the 74 patients. Three patients had hallucinations possibly related to the trauma; *“smell of blood”* and *“smell of burned rubber”*. Two patients were described as having olfactory hallucinations, but these were not further described.

#### Tactile hallucinations (*n* = 6)

Tactile hallucinations were described in six (8.1%) of the 74 patients. Two patients had tactile hallucinations described as an “electricity-like sensation”, e.g. “*flashback from concentration camp, a sensation of shock/electricity through the body”*.

Other tactile hallucinations described were combined with other hallucinatory experiences, for example; *“sensation of the neighbour’s dog licking her hand. And the last 20 days had visits from a man who rapes her”*.

#### Persecutory delusions (*n* = 37)

Persecutory hallucinations or severe suspiciousness towards other people were described in 37 (50.0%) of 74 records. Ten patients were clearly paranoid, or the word paranoid was used in the psychiatric record to describe the patient. For example; *“very paranoid, she thinks the crisis-centre and her husband wants her money. Appears clearly paranoid and with disrupted personality. The crisis-centre has pushed her down the stairs”*. Some patients had fears towards their countries of origin; *“The patient feels watched and is afraid of being persecuted by the Iranian security service. Checks cameras, changes address and does not use his phone”.* Other patients (*n* = 19) had a sensation of being persecuted or that someone was following them on the streets, e.g.; *“have the sensation that the Iranian Security Service is watching him”* or a more diffuse sensation; *“the feeling that “someone” is after her”, “sensation of being persecuted”*, six patients had possibly persecutory experiences or behaviours; *“don’t want to call home because he knows all international calls are being tapped”* or more clear persecutory delusional behaviour during the consultation; *“the door to the consultation room is open, the patient turns towards the door constantly, he cannot explain why”*.

#### Other psychotic features

Four patients were described as having delusion of control or possibly dissociative experiences; *“delusions of control where her legs were walking with her”* or *“sometimes have episodes where he doesn’t know who he is, where he is or what he is doing. Can suddenly sit and cut in himself and once he sat in a tree”.*

One patient was described as having a more bizarre delusion; *“periodically compromised reality testing and developed delusional ideas about a dead man in the mound close to the family’s home”*.

#### Reality testing

It was not always clearly displayed in the psychiatric records whether reality testing was preserved or not. Often, symptoms were mentioned but not further described, for example; *“feels persecuted”* or *“hears voices mumbling”*. At other times, patients seemed to reveal more and more lack of reality testing during the treatment programme. E.g. one patient, in the first medical consultation, appeared to have preserved reality testing; *“feel persecuted, but know deep inside that it is only thoughts”*, and later; *“suspicious, feel diffusely persecuted. See two-three times weekly one of the perpetrators, smell blood”,* and in the end of the treatment programme; *“there has been burglaries at a neighbour’s house, at another neighbour the children were kidnapped”.* Only three patients were described directly as lacking reality testing. Nine of the included PTSD-SP patients were directly described as *“appearing with preserved reality testing”* or with a description that makes it probable that the patients had preserved reality testing; *“see father, know it’s not real”, “rejecting all hallucinations”.*

#### Re-experiencing symptoms (flashbacks)

Five patients in the PTSD-SP group had symptoms described as being directly connected to flashbacks e.g.; *“angry voices in connection with flashbacks”, “flashbacks with airplane noise”.* In three of the five patients’ psychiatric records, persecutory delusions were described as having no connection to flashbacks. In one patient, the secondary psychotic features were described as being connected to flashback in one medical consultation but not in another.

## Discussion

### Estimating the prevalence of PTSD-SP

In the present study at CTP, 40.9% (95% CI 33.7–48.1%) of patients with PTSD are estimated to suffer from PTSD-SP, which is a prevalence comparable with similar studies within the field [16]. Twenty-nine of 220 patients from the potential study sample at the CTP were excluded because of lack of discharge diagnosis. As the majority of these 29 patients were not part of the CTP target group (refugees with trauma-related psychiatric disorders) we expect it to have a limited impact when calculating the prevalence of PTSD-SP. Hypnagogic and hypnopompic hallucinations were not included in the study definition of PTSD-SP. It is approximated that five patients have been omitted on this basis. The attempted exclusion of hypnagogic/hypnopompic hallucinations might lower the prevalence estimate of PTSD-SP slightly. On the contrary, the inclusion of patients with intact reality testing might increase the prevalence estimate of PTSD-SP in this study compared to others.

### Comparing the PTSD-SP group with the PTSD group

The discussion of whether PTSD-SP should be included in the PTSD diagnosis has been ongoing. Several studies are supporting the notable delineation of PTSD-SP from other psychiatric syndromes, as well as the association between PTSD and psychotic symptoms [16, 18, 34]. Critics of PTSD-SP as a diagnostic entity focus on the immense impact of PTSD patients comorbid diagnoses [35]. The present study suggests that PTSD patients differ as to complexity of symptoms and it is possible that a distinction in the diagnostic system could be reflected in an increased effort in the search for effective treatment for those patients with the most complex symptomatology. The following sections provide a description of why we find it possible, but challenging, to distinguish PTSD-SP from PTSD with comorbid diagnoses.

#### PTSD-SP vs. PTSD with comorbid depression and psychotic symptoms

We argue that PTSD-SP differs from PTSD with comorbid psychotic depression in several ways. In this study, a comparison of the PTSD-SP group with the PTSD group showed no statistical significant difference with regard to comorbid depression. Therefore, our results do not support the assumption that PTSD-SP is more often associated with major depressive disorder than PTSD, as assumed in earlier studies [16]. There is a very high prevalence of depression in both groups, a factor that can possible diminish differences when comparing them.

Tracing out the difference in symptomatology is another way of distinguishing PTSD-SP from PTSD with co-morbid depressive disorder and secondary psychotic symptoms. Psychotic symptoms seen in patients with depressive disorder tend to have a mood-congruent appearance whereas the symptoms seen in the PTSD-SP populations only rarely have a mood-congruent theme according to both Braakman et al. and this study. In this study we found only three patients in the PTSD-SP group with mood-congruent psychotic symptoms. The treatment implications from these preliminary findings need to be explored in future research studies.

#### PTSD-SP versus enduring personality change after catastrophic experience

Norredam et al. hypothesise that PTSD symptoms like flashbacks, hyper vigilance and increased alertness may slide into a more manifest paranoid state. This assumption is supported by the observations of this study as enduring personality change after catastrophic experience (DF62.0) was more prevalent in the PTSD-SP group than in the PTSD group. This finding could argue for the existence of a protracted severe subtype of PTSD. Scott et al. point to a possible dose-response relationship between traumas and delusions; the more traumas, the more delusions [18]. This study suggests that the features of trauma might be relevant to the development of delusions as patients exposed to torture and imprisonment had secondary psychotic features.

#### PTSD-SP vs. PTSD flashback symptoms or dissociative experiences

At this point, uncertainty remains as to whether it is possible to distinguish PTSD-SP from PTSD flashbacks or dissociative symptoms. The reason is partly that these symptoms are quite differently defined across different studies.

Our study tries to distinguish the differences with a descriptive approach and with broad inclusion criteria. In this way, numerous symptoms are included. The method is challenged by, for example, inter-interviewer differences since the psychiatric records are written by seven different medical doctors.

In Gaudiano et al.’s study, psychotic symptoms related to the PTSD patient’s traumas are categorised as flashbacks [35]. Contrary to this, hearing voices in PTSD patients is described as a part of dissociative experiences in a study by Brewin et al., which found that hearing voices occur in combination with other dissociative experiences on the Dissociative Experience Scale (DES) [36, 37].

It could be interesting to streamline the assessment of PTSD symptoms more within the research of the field, so a comparability of results is facilitated. Further studies, including studies of phenomenology, are needed to distinguish flashbacks from psychotic symptoms and dissociative experiences. This could lead to better clinical assessment of the symptoms in this category of patients, and facilitate a discussion about including psychotic symptoms in the PTSD diagnosis.

### Description of psychotic symptoms in the PTSD-SP group

Like Braakman et al., we found that symptoms in patients with PTSD-SP are primarily hallucinations and persecutory delusions [16]. In this study, it is not directly analysed whether the patients’ secondary psychotic features were connected to their previous traumas, as this was not directly specified in the psychiatric records. However, in many psychiatric records it appears plausible that the psychotic symptoms experienced by the patients refer to earlier traumas. This tendency is illustrated in the description of symptoms in the PTSD-SP group and supported by the series of case reports from CTP published in 2011 [17].

As reported in Braakman et al., bizarre behaviour and formal thought disorder were uncommon among PTSD-SP patients treated at CTP. Only one patient had more bizarre delusional experiences, which is compatible with the fact that only three patients in the PTSD-SP group presented an F2x diagnosis in addition to PTSD. No patients showed signs of formal thought disorder. It is not surprising that F2x diagnoses were rarely seen in the PTSD-SP population as an F2x diagnosis is normally an exclusion criteria at CTP.

### Strengths and limitations

This is an important study in an area with limited amount of studies conducted. The vast majority of studies within the field are conducted on war veterans, and a few studies on victims of sexual assault, whereas refugees as a group of investigation have received very limited attention.

Considering the broad inclusion criteria, the population investigated is representative of a clinical trauma-affected refugee population. Therefore the results are probably generalizable to other clinical settings working with severely trauma-affected refugees.

Furthermore, this is a rather large study compared to similar studies within the field.

The first author retracted the data from the patients’ psychiatric records as the only data collector. In case of doubt the data collector consulted the co-authors. Both the second and third authors were however clinicians at the CTP at the time and had clinical responsibility for patients in the study. It was therefore not considered appropriate that they would participate as second or third assessors. Unfortunately there were no other available, qualified and independent clinicians although this would have improved the method of this study.

The data collection was carried out retrospectively and the assessor was therefore totally dependent on the quality of the content in the psychiatric records. The psychiatric records presented great diversity as they were written by seven different medical doctors. Some psychiatric records were very descriptive, whereas others were brief. There seemed to be assessment diversity among the doctors concerning categorisation of the symptoms as either flashbacks or secondary psychotic features. This can have led to a selection bias in the dataset as only flashbacks described as being connected to psychotic or psychotic-like symptoms were included in this study’s definition of PTSD-SP. This choice was taken before study start in order not to overestimate the number of secondary psychotic symptoms.

Whilst collecting the data, it was a challenge to distinguish between hypnagogic/hypnopompic hallucinations and hallucinations experienced in wakefulness. Obstacles included the lack of description in the psychiatric records and the fact that numerous patients at CTP suffered from sleeping problems, which meant that patients were frequently awake for several hours during night-time. Sleep disturbance is, however, a very commonly experienced symptom among PTSD symptoms and this problem is therefore not limited to the present study, but poses an equal challenge in similar studies as well as in the treatment of this patient group. In summary, some of these challenges call for clear definitions and common articulation for the purpose of describing these phenomena.

The heterogeneity of the population was another obstacle. Nonetheless, the heterogeneity, as well as the inclusion of all patients admitted during a specific time period, makes the population generalizable to similar trauma clinics for refugees in Western countries. There might be cultural differences in describing and interpreting symptoms and experiences as the patients in the studied population had a large variety of cultural backgrounds. In addition, many consultations at CTP are dependent on interpreters, a step which can potentially lead to misunderstandings. Furthermore, both males and females were included in the population, and with an age span from 19 to 70 years.

Compliance problems might lead to skewing the results as a hypothesis could be that the worst functioning patients, like patients with secondary psychotic features, might have the most profound problems with treatment adherence. This might decrease the amount of psychotic symptoms found in the patient group. The problem is, however, negligible in comparison to studies concerning medical treatment given the fact that the patients ought not to complete the treatment programme in order to be included in the study. As described above there are several methodological limitations to this study. The most important is probably the retrospective data collection relying on the clinicians´ description of psychopathology. The implication of these limitations is a limited psychopathological description as to content and insecurity regarding the frequency. Nevertheless, keeping in mind the conservative approach when evaluating psychotic symptoms, the large amount of data collected in this consecutive sample of trauma-affected refugees brings new knowledge on the complexity of the psychopathology. This knowledge is believed to be useful for clinicians in the diagnostic process as well as for researchers when investigating effective treatments for this patient group.

## Conclusion

This study shows that psychotic or psychotic-like experiences are prevalent in the present trauma-affected refugee population and that the estimated prevalence of PTSD-SP is at least as high as in previously studied PTSD populations. It also suggests that severe interpersonal traumas such as torture and imprisonment are linked to an increased frequency of PTSD-SP.

The study provides a description of the psychotic symptoms and these results support that trauma-affected refugees suffer from a complex psychiatric condition. Furthermore, the psychotic symptoms described among these trauma-affected refugees seem to differ from symptoms of psychotic depression, which has consequences for treatment choice.

Whether PTSD-SP should be included as part of the PTSD diagnosis cannot be established on the basis of this paper. Nevertheless, the study should raise important awareness of that PTSD patients can have a very complex psychiatric condition, and that all aspects of their condition should be covered during assessment in order to ensure that the best possible treatment is offered.

The authors call for attention to psychotic symptoms in PTSD patients in order to improve documentation and understanding of the condition in both trauma-affected refugees and other survivors of trauma. As seen in both this study and in clinical settings, it is difficult to distinguish between symptoms like flashbacks, psychotic symptoms and dissociative symptoms. A more thorough understanding is needed to optimise diagnosis of refugees, which can prospectively contribute to improved treatment options for PTSD patients.

## Abbreviations

CTP: Competence Centre for Transcultural Psychiatry
DES: Dissociative Experience Scale
PTSD: Post-Traumatic Stress Disorder
PTSD-SP: Post-Traumatic Stress Disorder with Secondary Psychotic features
SCAN: Schedules for Clinical Assessment in Neuropsychiatry
